# Interactions of climate, socio-economics, and global mercury pollution in the North Water

**DOI:** 10.1007/s13280-018-1033-z

**Published:** 2018-03-07

**Authors:** Rune Dietz, Anders Mosbech, Janne Flora, Igor Eulaers

**Affiliations:** 1 Arctic Research Centre, Department of Bioscience, Aarhus University, Frederiksborgvej 399, 4000 Roskilde, Denmark; 2 Department of Anthropology, University of Copenhagen, Øster Farimagsgade 5, 1353 Copenhagen K, Denmark

**Keywords:** Biomagnification, Human exposure, Mercury, North water, Risk assessment

## Abstract

Despite the remoteness of the North Water, Northwest Greenland, the local Inughuit population is affected by global anthropogenic pollution and climate change. Using a cross-disciplinary approach combining Mercury (Hg) analysis, catch information, and historical and anthropological perspectives, this article elucidates how the traditional diet is compromised by Hg pollution originating from lower latitudes. In a new approach we here show how the Inughuits in Avanersuaq are subject to high Hg exposure from the hunted traditional food, consisting of mainly marine seabirds and mammals. Violation of the provisional tolerably yearly intake of Hg, on average by a factor of 11 (range 7–15) over the last 20 years as well as the provisional tolerably monthly intake by a factor of 6 (range 2–16), raises health concerns. The surplus of Selenium (Se) in wildlife tissues including narwhals showed Se:Hg molar ratios of 1.5, 2.3, and 16.7 in muscle, liver, and mattak, respectively, likely to provide some protection against the high Hg exposure.

**Electronic supplementary material:**

The online version of this article (10.1007/s13280-018-1033-z) contains supplementary material, which is available to authorized users.

## Introduction

Mercury (Hg) and in particular methyl-Hg biomagnifies and is present throughout the Arctic food chain, leaving high trophic marine avian and mammalian species with high body burdens ( Dietz et al. 1996; AMAP 1998). An estimated 200–300 tons of Hg are transported annually to the Arctic by long-range atmospheric and oceanic processes and ocean currents from various anthropogenic activities at mid-latitudes, emphasizing the worldwide nature of Hg pollution (AMAP 1998, 2011; Berg et al. 2001; Lindberg et al. 2001; Lu et al. 2001). A recent focus on Integrating mercury research and policy in a changing world” has just been issued in a special section on mercury in *Ambio* addressing the extensive global problems and effects from use, emission, and global transport of Hg across the World (e.g., Chen and Driscoll 2018). Although Hg is a naturally occurring element, worldwide anthropogenic activity has led to a manifold increase of Hg in the Arctic environment compared to pre-industrial times (Dietz et al. 2006, 2009; Braune et al. 2011). Furthermore, Hg emissions are projected to further increase unless new pollution abatement technologies are applied to coal-fired power plants (Streets et al. 2009).

A review on the literature on ancient temporal trend studies revealed that Hg concentrations have increased almost 20-fold since the industrial beginning in 1850 (Dietz et al. 2009). These observations have been confirmed by an updated study on polar bears *Ursus maritimus* for the North Water Polynya (*Pikialasorsuaq*, hereafter referred to as NOW; Dietz et al. 2011). Studies on soft tissue consumed by the Arctic Inuit populations are, however, less clear. Rigét et al. (2011) applied a statistically robust method to 83 time-series of Hg in Arctic biota collected over the past 2–3 decades. However, no consistent trend was evident across tissues and species from the circumpolar Arctic during the investigated period, most likely due to the fact that most time-series were too short to detect significant trends. There was, however, a tendency towards increasing Hg concentrations in marine species in Northern Canada and West Greenland compared to the North Atlantic Arctic. Though, data are too meagre to establish secure time trends for all consumed species in communities situated on both sides of the NOW, as well as other Arctic areas.

In addition to Hg, an array of chlorinated, brominated, and fluorinated persistent organic pollutants (POPs) have been transported to the Arctic and have been studied with respect to their effects, geographical and temporal trends, and human exposure. The NOW is, however, not the highest exposed site for these contaminants, and are, therefore, not of particular interest in the present study (AMAP 1998, 2003, 2004, 2009, 2015; Dietz et al. 2013a, b; Letcher et al. 2010).

The awareness of the presence, environmental increase, and detrimental health effects of Hg has recently resulted in the Minamata Convention (2017), which intends to reduce the international production and use of Hg. This convention is similar to the Stockholm Convention on POPs, which aims to eliminate or restrict the production and use of POPs and has, indeed, led to a decrease of the environmental impact of these contaminants in the Arctic (Dietz et al. 2013a, b; Rigét et al. 2013, 2015; Stockholm Convention 2017). An urgent need for international mitigation of Hg is called upon by the alarmingly high Hg concentrations in local inhabitants of the Avanersuaq region of Northwest Greenland facing the NOW. The observed concentrations exceed 5–50-fold that of other Arctic Inuit populations, including the Iñupiat, Yup’ik, Dene/Métis and other indigenous and non-indigenous Arctic populations (AMAP 1998, 2009). These studies also identify the Avanersuaq population, i.e., the Inughuit, to be the highest exposed population within Greenland. Therefore, we investigated the human exposure to Hg in Avanersuaq using a cross-disciplinary approach combining data on chemical contaminants, catch information, historical, and anthropological perspectives. We first aimed at elucidating to which degree the traditional food is being compromised by Hg pollution, and, second, how seasonal and inter-annual hunting and dietary patterns may be tied to a changing health risk.

## Materials and methods

### Subsistence hunt and dietary choice

The extensive amount of hunting data for a wide range of hunted species in the period 1993–2013 collected by the Ministry of Fisheries & Hunting, Government of Greenland (Piniarneq 2015) was used in a new approach to calculate the amount of Hg entering monthly and annually the hunting societies around the NOW. The average meat outcome per species was on average estimated at 30% of the average total weight of the hunted species. We hence used this as a crude estimate of ingested meat from the hunted species obtained from the *Piniarneq* database, which does not hold information on size, age, and sex of the hunted individuals, known to have an effect of the weight of the hunted wildlife. Only meat was used in the exposure models as the majority of the other edible parts, i.e., the majority of blubber, lungs, spleen, liver, kidneys, and intestines, are consumed by the sled dogs. The introduction of quotas and seasonal hunting regulations, as well as new fishing opportunities and hunting limitations from climate related unfavourable ice and weather conditions, means that the hunting statistics summarizes more than the animals’ presence in the area, but also reflects complex changes over time. Demographic data on population size, sled dog numbers, trade, economic, and occupational data were obtained from Greenland Statistics (2017) for Avanersuaq, and the periods were dependent on the available data and questions discussed. In addition to this, interviews and general anthropological conversations with the local population contributed to the general picture described below. Ethnographic fieldwork concerning the local distribution and symbolic, cultural, and monetary values attached to food was carried out in during 2014–2016 primarily in Qaanaaq and Savissivik using participant observation and unstructured interviews.

### Mercury exposure

Data on meat Hg concentrations were not available for each species or year during the 20-year period spanning 1993–2013. The Hg data set used in the present study was, therefore, either extracted from published literature or analysed from freshly collected meat, i.e., muscle samples conducted under the NOW project. The chemical analysis for Hg followed previously reported methods of element extraction (Søndergaard et al. 2011; Sonne et al. 2014). Each muscle sample was freeze-dried and homogenized, while the remaining tissues were analysed as such. For each sample, the dry weight content was gravimetrically determined and a subsample was subjected to microwave digestion in 8 mL of a HNO_3_:MilliQ water mixture (1:1, *v:v*). The digested solutions were diluted with MilliQ water and analysed for a wide range of trace elements, though only Hg data are employed within the present study. An Agilent 7900 CE ICP-MS was used for quantification at the accredited trace element laboratory of the Department of Bioscience of the Aarhus University, Denmark.

Temporal trend analyses of the hunted game over time were performed using R (R Core Team 2016). Prior to the statistical analyses, individual hunted numbers were log-transformed to obtain normality and homoscedasticity of the variance. To test for temporal trends, linear regression analyses were applied using individual log-transformed hunted wildlife numbers as the dependent variable and year as the explanatory variable (e.g. Dietz et al. 2004, 2011).

**Fig. 1:**
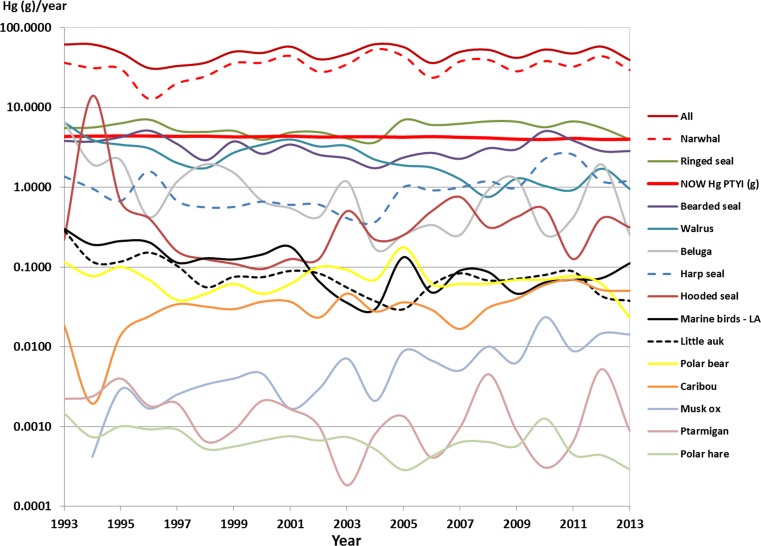
Temporal Hg load in the hunted game of Avanersuaq based on average hunt from 1993 to 2013 (Piniarneq 2015) and average Hg loads in muscle tissue from the published and unpublished contaminant studies in Greenland (see Table S3 for details)

The yearly average Inughuit Hg exposure, expressed per gram meat (g), as a result of the hunted game in Avanersuaq over time was calculated by combining data on average meat intake from the hunt from 1993 to 2013 (Piniarneq 2015; Electronic Supplementary Material, Table S2) with data on average meat Hg concentrations taken from published and unpublished contaminant studies in Northwest Greenland (Fig. 1; Table S3, S4). To link the yearly Hg intake to guideline values, the Provisional Tolerably Yearly Hg Intake (PTYI) and the Provisional Tolerably Monthly Hg Intake (PTMI) for the community, assuming an average individual body weight of 60 kg, were calculated according to the following (US Environmental Protection Agency 1997):

$$ {\text{PTYI }} = {\text{Hg }}\left( {\text{g}} \right) \times 52   {\text{weeks}} \times {\text{population number}} $$

and

$$ {\text{PTMI }} = {\text{Hg }}\left( {\text{g}} \right) \times 4   {\text{weeks}} \times {\text{population number}} $$

for which the mean Inughuit population size is 849, varying from 795 to 881 between 1993 and 2013.

## Results and discussion

### Long-term changes in the hunt, diet, and Hg exposure

**Fig. 2:**
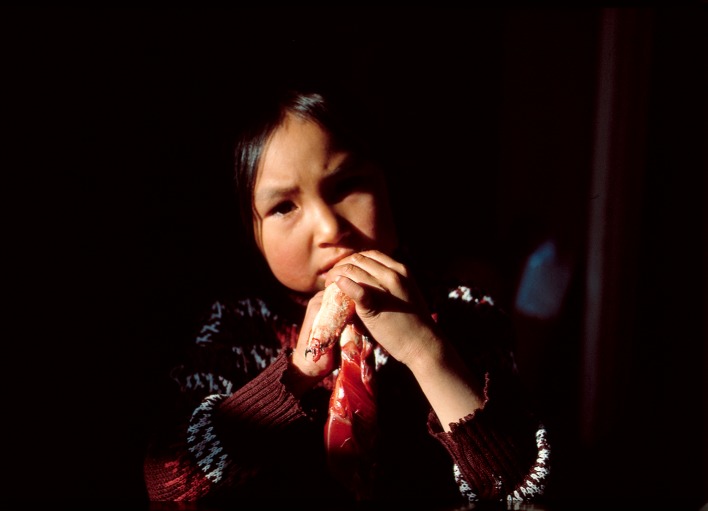
Little auks are the most numerous birds eaten, but due to their small size, they range as number 10 in importance as food resources in the North Water (Photo: Rune Dietz)

**Fig. 3:**
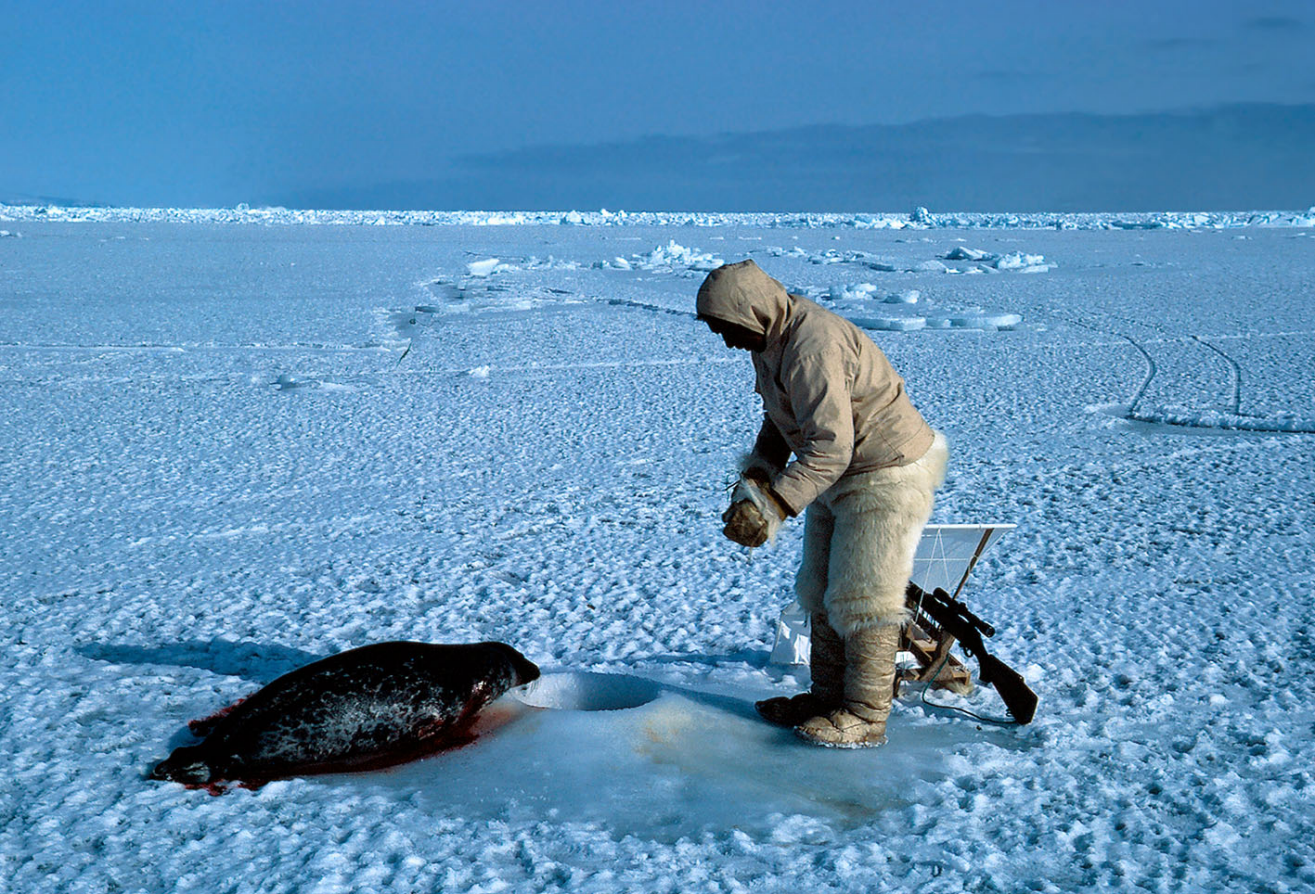
Ringed seals are one of the most important food resources in the North Water as it is present and can be hunted year-round (Photo: Rune Dietz)

**Fig. 4:**
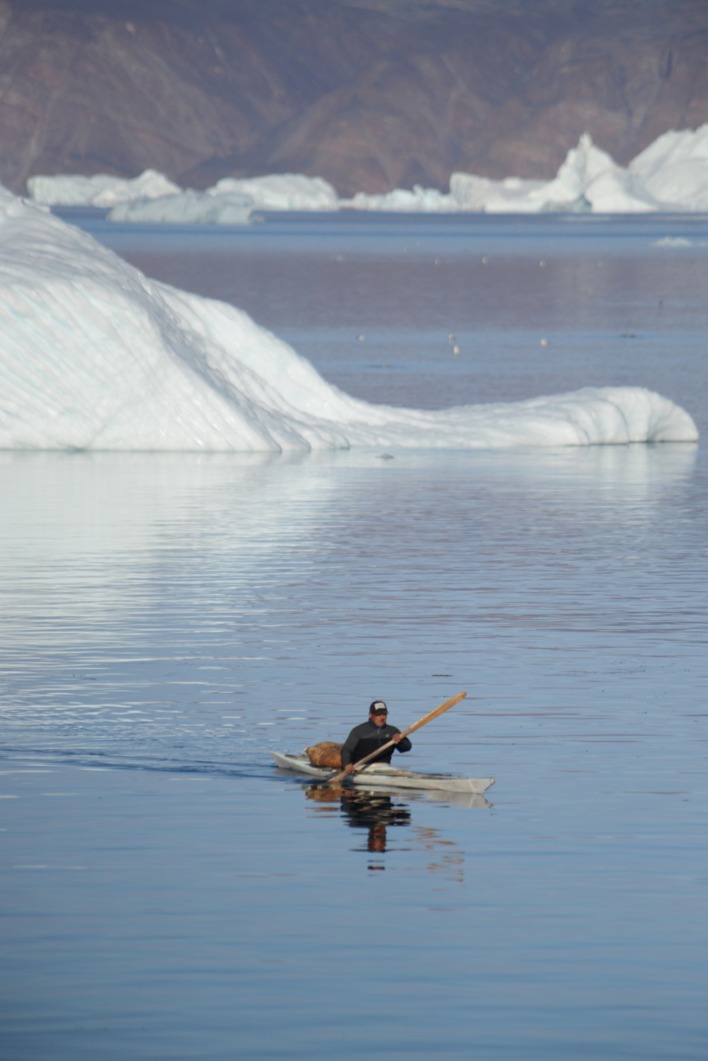
Narwhals hunted in Avanersuaq are hunted from kajak, where the whales are harpuned prior to being shot. This traditional hunting method results in practically no hunting loss (Photo: Rune Dietz)

**Fig. 5:**
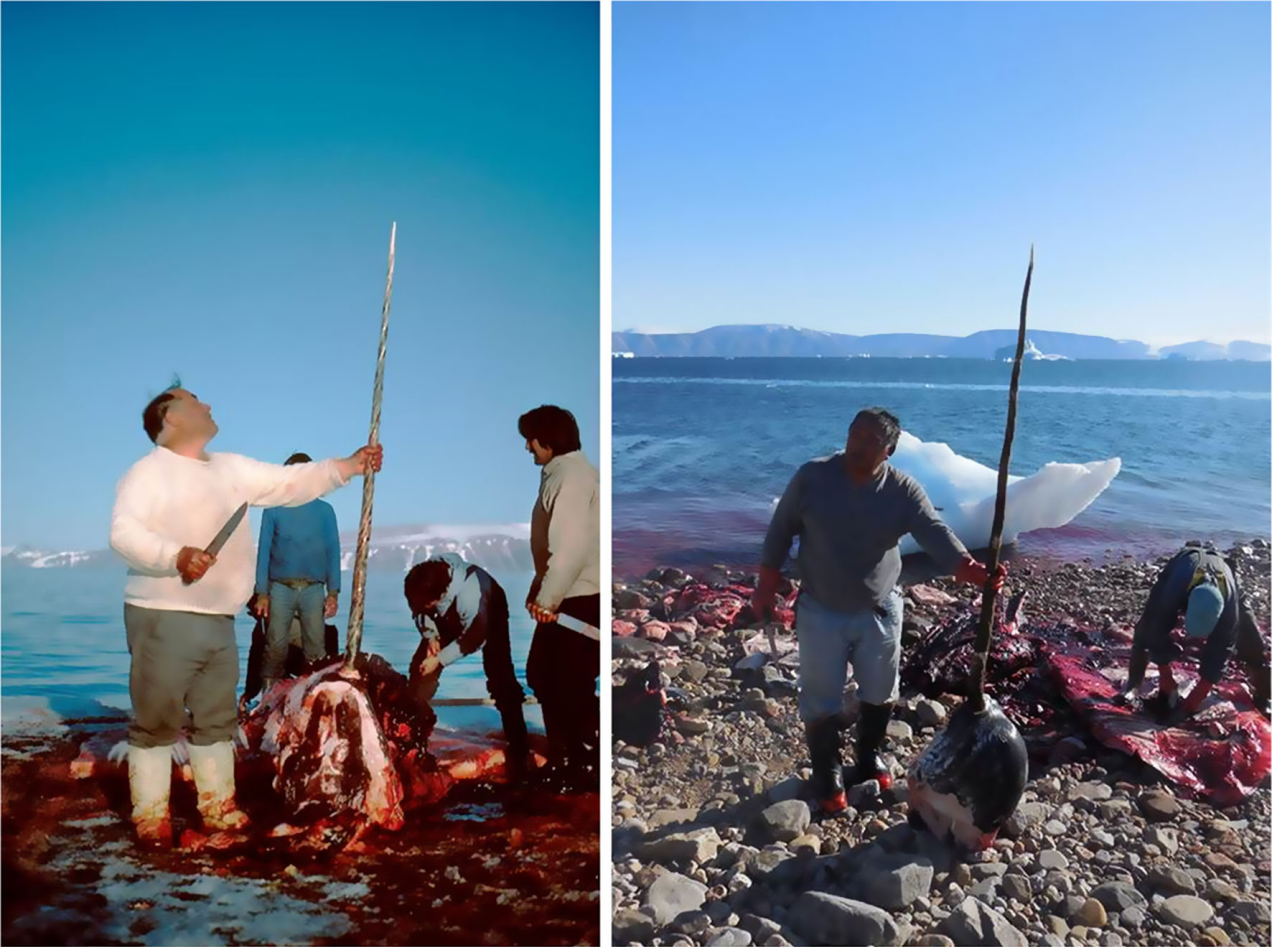
In addition to the meat and mattak, narwhals are hunted for there valuable tusks. The two pictures were taken 31 years apart. To the left, Massauna Kristiansen is looking at the tusk, while his sons are flensing the narwhal caught in June 1984. To the right, the son Mamarut Kristiansen is engaged in narwhal hunting following the methods learned from his farther in August 2015 (Photos: Rune Dietz)

Between 1993 and 2013, hunters in Avanersuaq caught an average of 27 925 animals each year (Fig. S1, Table S1). In absolute numbers, the top five (in declining order) are composed of little auk *Alle alle* (Fig. 2; 22 599 individuals or 81%), ringed seal *Pusa hispida* (Fig. 3; 2704 individuals or 10%), Brünnich’s guillemot *Uria lomvia* (1200 individuals or 4%), Arctic hare *Lepus arcticus* (287 individuals or 1%), and bearded seal *Erignathus barbatus* (178 individuals or 1%; Table S1). Considering meat to be on average 30% of a mammal’s mass, these hunting numbers amount to an estimated 287 t in total meat consumed (Table S4). Moreover, roughly, a similar amount of blubber, liver, kidney, heart, and intestines is estimated to be consumed by the hunters’ sled dogs. From a mass perspective, the yearly input of meat is mostly composed of ringed seal (27 t), followed by narwhal *Monodon monoceros* (Figs. 4, 5; 19 t), walrus *Odobenus rosmarus* (15 t), bearded seal (9 t), and beluga *Delphinapterus leucas* (3 t) (Fig. 1; Table S2).

For most of the species, the hunt shows yearly fluctuations without any noticeable trends over the data period. However, the catch percentage of walrus, beluga, Arctic hare, little auk, common guillemot *Uria aalge*, common eider *Somateria mollissima* and black-legged kittiwake *Rissa tridactyla* dropped significantly by 4–11% year^−1^ over the past 2 decades, while the catch percentage of muskox *Ovibus moschatus*, caribou *Rangifer tarandus,* and harp seal *Pagophilus groenlandicus* increased significantly by 8–14% year^−1^ (Figs. 1, S1, S2).

There is quite a large difference in personal preference for what part of the animal body is consumed, depending on the area of Avanersuaq, the season, the species, the hunter, and, of course, the occasion. Conversations with the local population indicate that certain organs such as liver and kidney may be less popular than they used to be, and that it is primarily the older generations who continue to eat these at the present day. We do note, however, that some local specialities have organs as their central ingredients, and these tend to be eaten by most. Some species such as bearded seal and polar bear *Ursus maritimus* liver are, however, never consumed due to their toxic vitamin A load.

It is well documented that the majority of Hg in meat is present as methyl-Hg (Dietz et al. 1990; Wagemann et al. 1998), a Hg species readily (95%) taken up over the intestinal wall and, therefore, toxic to mammals (Berlin 1986; WHO 1993; Mori et al. 2012; Dietz et al. 2013c). Hence, meat represents the primary source of human Hg exposure associated to consumption of wildlife, representing up to 70% of the Hg taken in by consumption of various tissues including liver, kidney, heart, intestine, and blubber (Dietz et al. 2017). With the estimated average yearly influx of 46 g Hg year^−1^ (range: 33–62 g Hg year^−1^), the PTYI was on average exceeded by a factor of 11-fold (range: 7–15 fold; Table S4). This transgression would be even higher if other tissues from these marine mammals and birds, from fish, and from other non-recorded items had been included.

### Seasonal changes in the hunt, diet, and Hg exposure

**Fig. 6:**
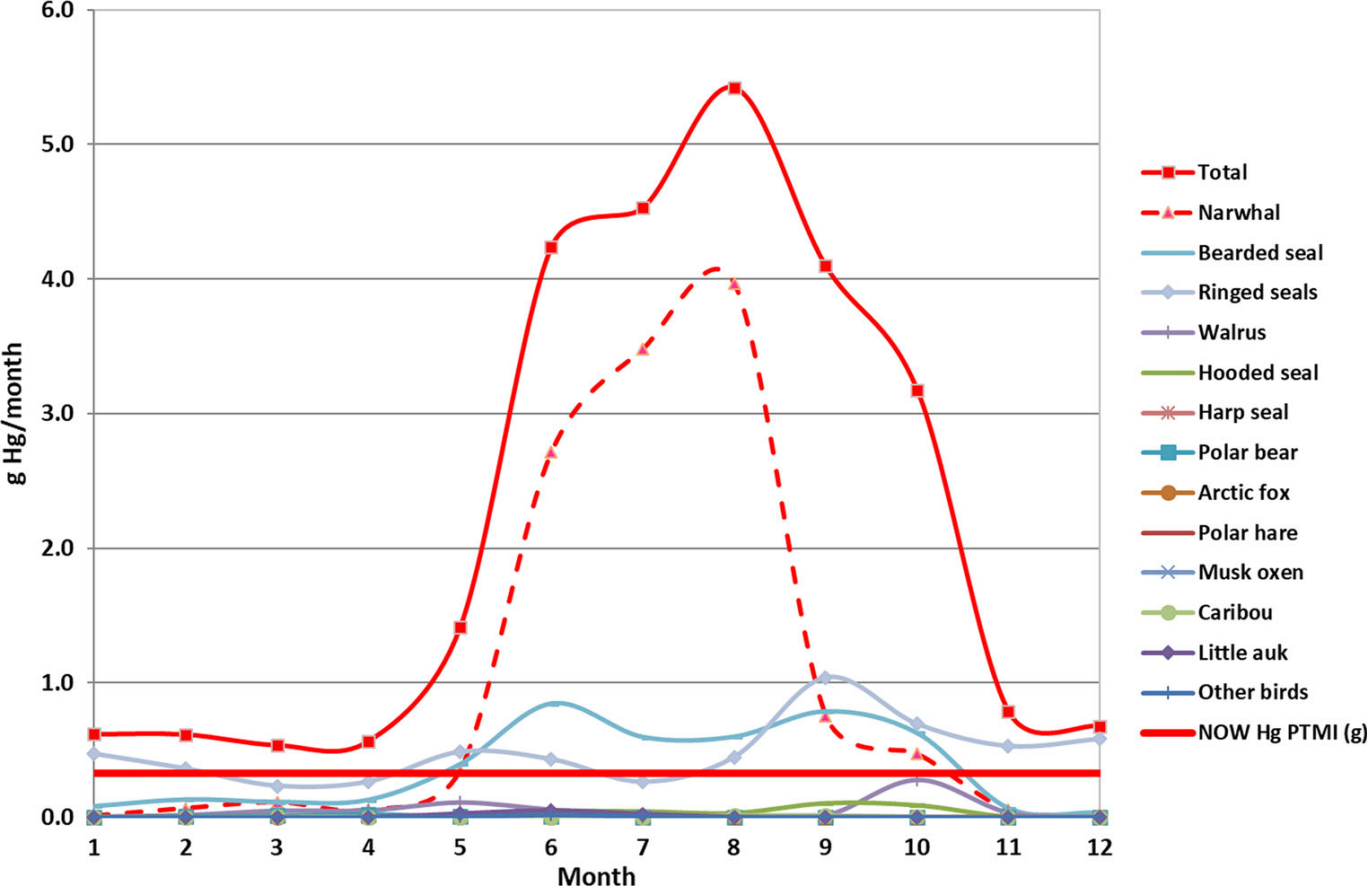
Seasonal Hg load in the hunted game of Avanersuaq based on average hunt from 1994 to 2014 (Piniarneq 2015) and average Hg loads in muscle tissue from the present study and published and unpublished contaminant studies in Greenland (see Table S5 for details)

The marked seasonal environmental variation, represented by the extent of land fast ice, impacts also the presence and accessibility of animals (see also Flora et al. 2018). During autumn and winter, most birds will migrate southwards, which also applies to harp and hooded seals, the majority of narwhals and belugas, as well as baleen whales (Vibe 1950; Heide-Jørgensen et al. 2003; Dietz et al. 2008; Andersen et al. 2009; CAFF 2013; Grønnow 2016). From the hunt numbers reported by Piniarneq 2015, it becomes evident that the major Hg influx occurs during the 5 months from June to October, during which narwhals are present in the region and are the dominant caught species (73%). Their presence essentially drives this seasonally peaking influx of Hg into the Inughuit population as the majority of the meat is eaten fresh and only minor proportion is dried or frozen (Fig. 6, Table S1). Hence, hunted food is mainly eaten at the season when it becomes available as it provides a welcomed variation in the food intake. Actually what characterises the so-called “hunter-gatherer cultures” is that they do not have food storage in the same way that agricultural societies do. Nor is it the case that Inughuit preserve food “for the long and dark winter”. Hunting and fishing for human and dog consumption continue throughout the year. Although Inuit throughout the Arctic do have varying ways of preserving food for the most part dried meat and fish, frozen or fermented, are treated as light delicacy meals usually consumed at special celebrating occasions. Hence, the estimated average seasonal monthly influx of 2.1 g Hg month^−1^ (range: 0.53–5.2 g Hg month^−1^) resulting from the hunted and consumed wildlife meat results in the PTMI on average to be exceeded by a factor of 7-fold (range: 2–16 fold; Fig. 6; Table S5). This seasonal pattern is supported by a pilot study on seasonal exposure of the Inughuit population based on weekly samples of facial hair from local inhabitants with different occupation and from different parts of Avanersuaq (Dietz et al. 2017). These results likewise show a higher load during summer, beginning to increase in June and peaking in August–September after which the exposure declines due to reduced narwhal consumption and consequent Hg excretion. One of the occupational hunters analysed showed up to 100-times larger Hg concentrations than reference persons, transgressing up to 45-times the US Environmental Protection Agency guideline value for acceptable Hg concentration in human hair.

**Fig. 7:**
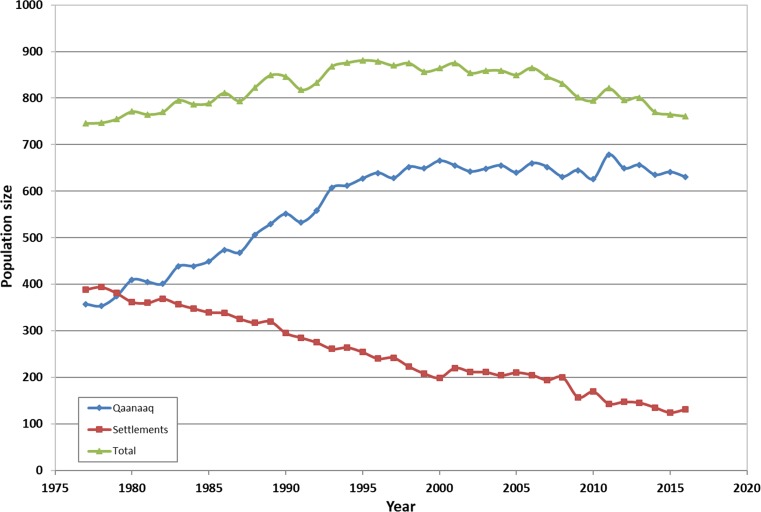
Qaanaaq human population over the last 4 decades presented for Qaanaaq versus settlements (Greenland Statistics 2017)

The above meat-orientated estimate alone is certainly an underestimation of the exposure to the hunters as also blubber, and for a part of the population also liver, kidney, and intestine are being consumed. As the yearly amount of meat on average totals around 86 t over the last 2 decades, the total edible part of the wildlife (edible non-meat likewise composes ca. 30%), including the meat influx, is likely closer to 172 t year^−1^. However, a substantial amount is also needed for feeding sled dogs (see discussion below). How much local food is eaten per hunter per day is uncertain as the literature values range from a rather low estimate of 169 g day^−1^ (calculated for the entire Greenland; *n* = 2245 between 2005 and 2008) to a rather high estimate of 943 g day^−1^ (for the Belcher Island region for 1995–1996; Wein et al. 1996; Jeppesen et al. 2012). We estimate the average daily intake for Avanersuaq to be close to 500 g day^−1^ and would lead to a yearly wildlife demand of 155 t year^−1^ for the average Inuight population (Fig. 7). This is considerable more than our estimate of meat intake, estimated at 80 t, resulting from the wildlife hunt.

**Fig. 8:**
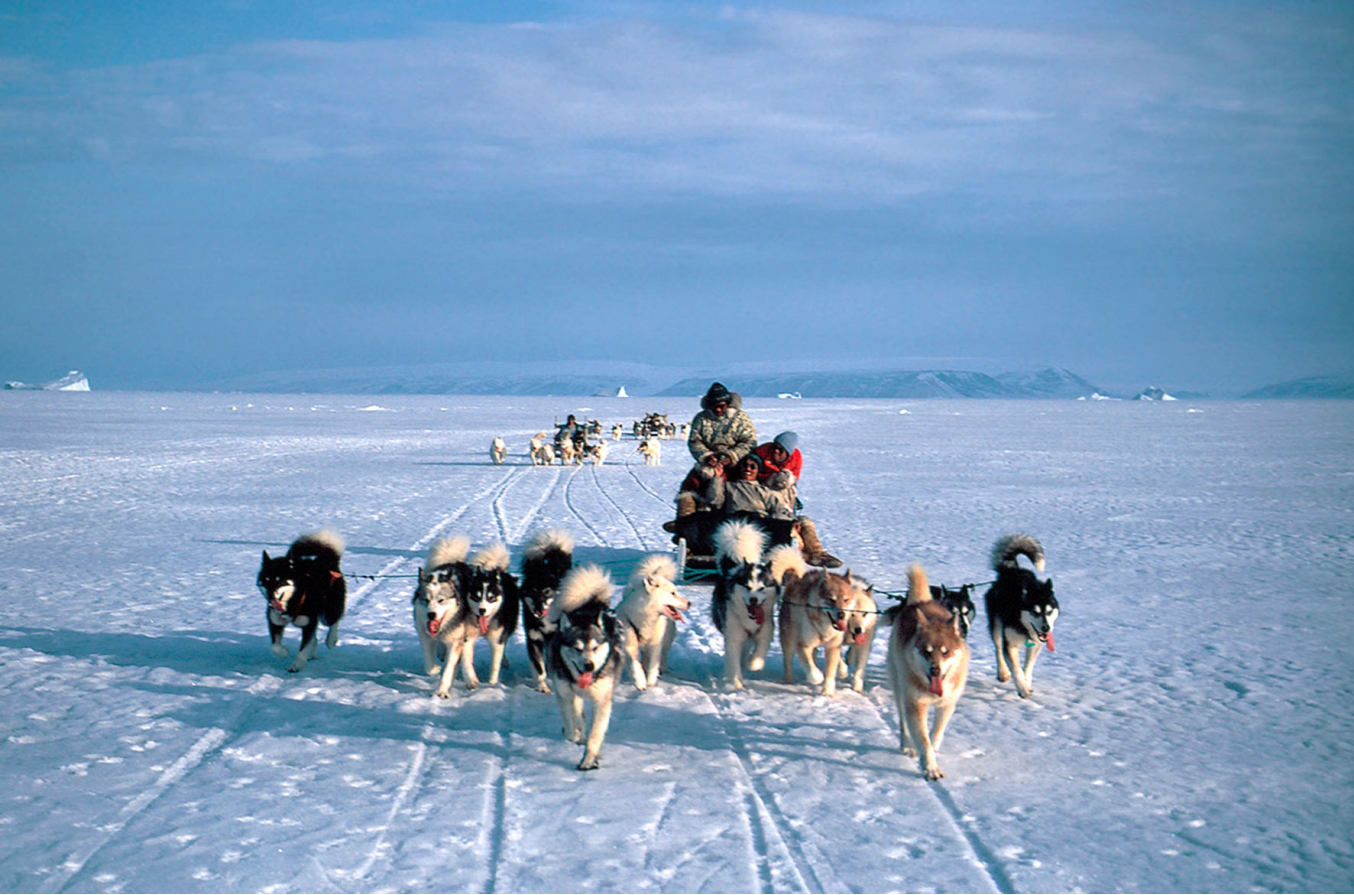
Dog sledge is and important during the winter hunt. However, the sled dog numbers have been declining all over Greenland including Avanersuaq due to less favourable ice conditions, longer open water seasons and the fact that the number of occupational hunters itself is in decline (Photo: Rune Dietz)

The fulltime hunters in Avanersuaq have some 1016 sledge dogs to feed (Greenland Statistics 2017). However, the number of sled dogs has been declining all over Greenland including Avanersuaq, partly due to less favourable ice conditions and longer open water seasons, and perhaps more crucially, owing to the fact that the number of occupational hunters is in decline (Fig. 8). In Avanersuaq, the sled dog numbers have significantly declined by 1.4% per year since 1993 with an average reduction of 39 dogs per year between 1990 (*n* = 1800) and 2016 (*n* = 1036), which is similar to the overall trend in Greenland where the decline has been 1.5% per year (Greenland Statistics 2017; Fig. S3). As the total volume of catch has not overall changed significantly, the reduction in the number of dogs is likely to increase the amount of food available for human consumption as the hunters have fewer dogs to feed. Dogs are fed different feeds and amounts, depending on the season and the intensity with which they have worked. According to Gerth et al. (2010), Greenland dogs are fed every second day or third day with seal, walrus, or whale meat during summer with a meal size of 1–2 kg meat per dog. In winter, these dogs receive melted and heated chunks of seal or walrus meat plus internal organs and tissue every other day (approx. meal size of 2 kg per dog). Only during hunting trips in winter, additional food may be provided to the dogs. A sled dog, therefore, consumes approximately 519 kg food per year (Gerth et al. 2010) which is slightly more than our estimates based on conversations with the local hunters. Hence, using an average of 500 kg per dog per year, the provisions for the 1036 dogs in the entire region amounts to as much as 518 t year^−1^. However, many hunters also buy food pellets in the local shop to secure provisions. Unfortunately, information on the amount of imported food over the time period dealt with in the present study was not available.

**Fig. 9:**
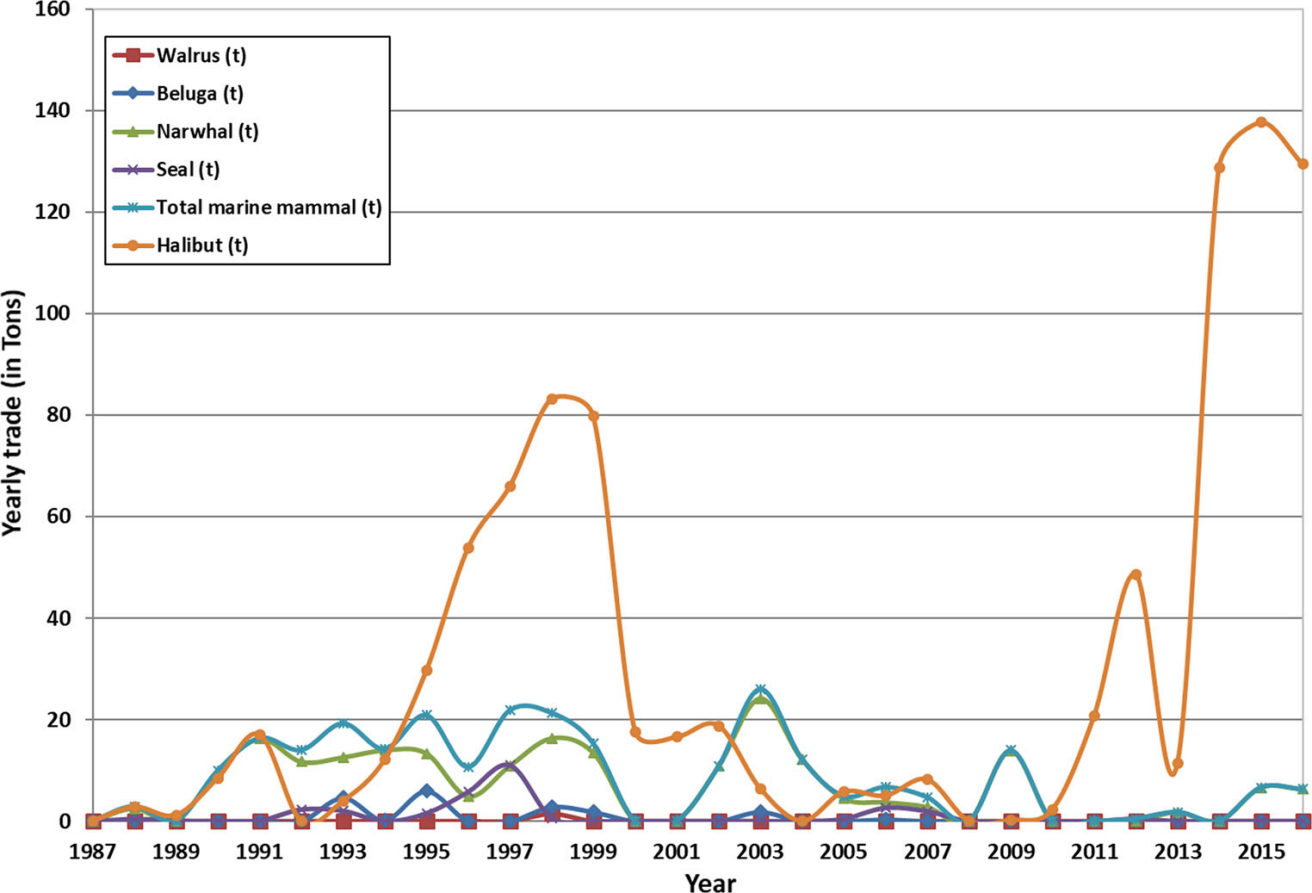
Yearly trade of wildlife and Greenland halibut fishery (in tons) to Inughuit Seafood and Royal Greenland in Qaanaaq over the last 3 decades (Greenland Statistics 2017)

Another factor that may affect the amount of local food available to the community is how much of the game is being traded into Royal Greenland and Inughuit Seafood in Qaanaaq established in 2014. Today, the mainstay trade is Greenland halibut *Reinhardtius hippoglossoides*. For marine mammal species, amounts vary substantially from year to year, while, on average, some 8.7 t are traded in per year over the period 1987–2016; Greenland Statistics 2017). Historically, the majority of this amount relates to narwhal products (81%), unspecified seal meat, not traded since 2007 (11%), and beluga products (6.8%), whereas walrus meat constitutes less than 0.6% owing to the relatively small quota and its main use to feed dogs (Fig. 9). As the majority of the narwhal and beluga products (88% or 7.7 t year^−1^) are their skin (*mattak*) which in itself has not a high Hg or POP load in contrast to the attached blubber (Dietz et al. 2004). This trade, therefore, reduces the healthy consumption of this important *mattak* resource.

Adding up the demand of the community (154 t year^−1^) and their dogs (515 t year^−1^), the total demand amounts to some 669 t year^−1^, which is approximately fourfold our estimated local catch of 172 t year^−1^. In addition to the game hunt, bird eggs are being collected but even with some 2000 eggs collected from different birds such as glaucous gull *Larus hyperboreus* and little auk this most likely amounts to less than 0.2 t year^−1^. On top of hunted catch, fisheries for Arctic char *Salvelinus alpinus* and sculpin *Myoxocephalus* spp. have always been common though may only be in the magnitude of 1.0 t year^−1^. However, during recent years, fishing has become increasingly important and long-line fishery for Greenland halibut has become a significant resource assuring 130 t of trade with Royal Greenland and Inughuit Seafood (Fig. 1). Sources of cash income have, indeed, changed in significant ways over the past decades (Flora et al. 2018; Hastrup et al. 2018). The cost of living is high, and with the hunting endeavour, itself being costly, the restrictions on the trade in by-products like tusks, furs, and to some extent seal skin have made the halibut a welcome source of income. The amounts of additional halibut as well as other fish species like Atlantic wolfish *Anarhichas lupus*, polar cod *Boreogadus saida*, spotted wolffish *Anarhichas minor*, Atlantic cod *Gadus morhua,* and redfish *Sebastes marinus* for private consumption is limited as they are not common or prized, and therefore, a little effort is allocated to such fishery. However, an estimate from hunter and municipality politician Jens Danielsen (pers. comm.) some additional 20 t may be realistic. The discrepancy between meat and fish derived food and the food demand are most likely due to a substantial consumption of imported food, an underreporting in the hunting statistics, an overestimate of the food demand of both humans and dogs, or a combination of these factors.

### Human exposure and food security

It is beyond the scope of this paper to summarize the human health effects of Hg as this is an extensive research area for medical doctors. Extensive human health assessments have though been reviewed under the Arctic Monitoring and Assessment Programme (AMAP) over the last 2 decades (AMAP 1998, 2003, 2009, 2015). Overall studies have generally shown associations with cardiac autonomic neuropathy indicating effects on the nervous system, whereas there have not been shown effects of Hg exposure or conflicting effects on blood pressure, cardiovascular diseases, and diabetes that pose severe threats elsewhere in the World (AMAP 2015; Larsen 2017). Over the recent years, there has been a growing recognition on taking genetic information into account when evaluating susceptibility to Hg-related health effects and various diseases, including fatty acid metabolism (AMAP 2015; Larsen 2017). Climate change is regularly seen to pose a threat to all of the World Health Organization’s three pillars of food security, namely, (1) the *access to food*, (2) the *availability of food,* and (3) *food use*, which relates to the harvesting, sharing and consumption of local foods (Paci et al. 2004; Wesche and Chan 2010). These are unique to the Arctic region in such a way that we may be able to speak of cultural food security (Power 2008). Narwhal is one of the foods in Avanersuaq that is hunted and shared in accordance with particular prescriptions, and is, therefore, intimately connected to human relatedness. Normally hunting and sharing narwhal are important for human kinship and relatedness. However, the contamination of food highlights a different aspect of food security relating to risk and safety. In Avanersuaq, it is not that foods are not available, nor that people cannot access these, but rather that food (understood broadly) is engulfed by boundless global uncertainties and safety issues through the risk of contamination (Hastrup et al. 2016). This applies to local and imported foods alike.

The presence of Hg and other biomagnifiable contaminants in marine mammals is an issue that has been widely discussed with varying intensity in Greenland for decades. It is generally seen as a product of global pollution that intrudes local staple foods and puts the health of local populations at risk. These debates are counterbalanced by other findings that obesity and other life style illnesses play a greater role in adverse health outcomes than exposure to contaminants (Bjerregaard and Mulvad 2012). These findings are echoed locally in Avanersuaq, where (as in the rest of Greenland) shop-bought foods or Danish foods (*Qallunaamernit*) are regularly juxtaposed to local foods (*Kalaalimernit*) in terms of physical and psychological health. We should note, however, that determining how people locally understand and act in terms of safe and healthy food categories is not a straightforward matter of local versus imported. Each are at different times embraced and rejected. Either way, the contamination and safe consumption of food is a problem of both global and local magnitude. It comes from the outside and seeps through food and the health of the population (Hastrup et al. 2016). In Qaanaaq, people are not blind to the fact that illnesses like obesity, diabetes, and cancer never used to be issues in the past, and that there may be a causal link between these illnesses, and the food people consume. Food safety and the human exposure to contaminants thus extends far beyond the Hg concentrations in marine mammals, and should be understood as connected (in cause and effect) to other issues relating to contamination, health, and food, including hunting practices, social relations, and societal subsistence.

Dietary preferences in Avanersuaq vary not only according to the seasons or when animals are hunted, but also among households and individuals. People here, as elsewhere, have different strategies for dealing with the risks related to food consumption. Some prefer to eat foods that have been harvested locally, while others may prefer shop-bought food. For some, food consumption is not a matter of choice, but of necessity and financial means. Some families cannot afford to shop for food, while others, who are in closely knit hunting families, cannot afford to purchase locally sourced foods as frequently as they may like to. Food security and safety, therefore, is also entwined with economy. In Denmark, food consumers handle food-related risks (additives, pesticides, growth hormones, etc.) in socially and politically complex ways (Halkier 2004). Some Danish consumers keep themselves informed and adapt their food preferences accordingly; others interchangeably ignore and adhere to health warnings in frustrated perplexity, while many assume a casual approach to food risks citing feasibility and pragmatism. What is necessary, safe, and preferred, is far from a universal matter. Many understood the Bovine spongiform encephalopathy crisis in Great Britain to be a health issue and, therefore, avoided British beef, while others regarded it a political vendetta against the British farmers and economy, and prioritised British beef over any other. A similar complexity is present among people in Avanersuaq, where the discourses about contamination of food interweave with notions of locality, freshness, and nutrition. What is regarded as nutritious 1 day may be regarded as contaminated the next, and people’s food choices thus relate as much to matters of health and what is preferred, as they do to what is feasible and available. Sometimes, contaminants are suspected to be prevalent in local foods, but the nature and frequency of the contamination is unknown. Other times certain foods are fiercely avoided, because people consider them to be contaminated due to observations of individual animals with strange growths or unusual appearances. In conversation, one occupational hunter stated that he deeply desired more information about how pollution was affecting his livelihood, i.e., local foods. Others, including the same hunter, also maintain that it is not the local foods that are unhealthy, but rather the poor quality of imported foods, which are anonymous in origin as well as ingredients, or, indeed, the mix between local foods and shop-bought foods. These foods, delicious as they may be, are commonly seen to lack nutrition and lack the capacity to satisfy hunger.

The way in which contamination seeps varies greatly. One example relates to the events by which a *kiviaq* that had been consumed in the village Siorapaluk in July 2013, tragically claimed the lives of two people. These examples remind the hunters of the fact that food can be poisoness as a result combination airtight conditions of a plastic bag together with natural occurring bacteria. Another three persons were rescued by a medical team flown in from Nuuk administrating trivalent antitoxin and mechanical intubated ventilation. The accident was understood to be caused by foodborne botulism (Hammer et al. 2015), arisen from the technique of fermenting little auks in seal blubber inside a tightly stitched sealskin and later stored in a plastic bag. Some tend to blame the fermentation practice alone, deeming it dangerous. Locally, however, the *kiviaq* is not unanimously understood to have claimed human lives due to the failure of an age-old practice; nor even the fact, that this particular *kiviaq* had been made, by thick-billed murres or eiders, which are significantly larger than the little auk and, therefore, require longer fermentation time. Rather, many locals continue to maintain, that the *culprit* was the plastic bag, which had been used to store the birds after the *kiviaq* had been opened; chemicals having seeped into the fermented birds. According to Hammer et al. (2015) foodborne botulism results from ingestion of toxin-contaminated food, the toxin being produced in an environment allowing the anaerobic, Gram-positive bacterium *Clostridium botulinum*, to grow, facilitated by anaerobic conditions in a plastic bag with low concentrations of salt, sugar, and acid. According to the literature outbreaks of botulism have previously been reported among the Inuit populations of Alaska, Canada, and Greenland (Miller 1975; Sørensen et al. 1993; Loutfy et al. 2003; Leth 2014). The tension between the explanatory frameworks is more than a noticeable, one attributing the food technique and knowledge itself in combination with the airtight conditions of a plastic bag, while the other places the contamination beyond the food itself, in a plastic bag that invisibly seeps toxins into age-old life sustaining food traditions. Food contamination may also be seen as firmly anchored within a particular locality, although also having arrived from the outside in form of the B52 bomber carrying four hydrogen bombs that crash landed at the Thule Air Base in 1968. Elderly Qaanaaq residents today, who witnessed the landing and accidentally inhaled smoke from the fire, ascribe their ailments and illnesses to these events. Today, some hunters refuse to hunt in Wolfstenholme Fjord (*Uummannap Kangerlua*), asserting that food from here is contaminated and damaging to health. Others continue to hunt in these fjords as they always have. A report into the health status in Avanersuaq (Bjerregaard and Dahl-Petersen 2010) prompted by the fact that the population expressed concern about the comparatively high occurrence of terminal illness, which they interpreted as caused by plutonium entering the food chain. The report concluded that neither the rate of death nor cancer (nor other Plutonium related illnesses) could be shown to be higher in Avanersuaq than in other parts of Greenland. Nor could the study show any marked difference in the concentration of POPs in the blood between the population in Avanersuaq and the rest of Greenland. What is crucial here then is the local concern about contamination and the overall associated health status, the rate of which the local self-assessment showed to be higher than in other parts of Greenland, thus underscores the notion of uncertainty that runs through food safety issues. The concentration of Hg in the blood among the participants, however, was found to be considerably higher than in the rest of Greenland. The report links this to the high consumption of marine mammals, narwhals especially (Bjerregaard and Dahl-Petersen 2010).

What is qualified locally as contaminated or safe is at one and the same time concrete and blurred, both in terms of how the contamination manifests, and in terms of how people orient themselves accordingly. The intake of Hg is marked by seasonal variations that go hand in hand with the migration of animals and hunting patterns. Hg concentration can, therefore, scarcely be said to be constant or universal, neither in narwhals, nor in humans. Instead, these will vary as hunters and their families orient themselves towards different resources in the hunting calendar. Although some may recognise the high levels of Hg present in narwhal itself, its *mattak* is widely recognised as a food source that contains some of the most vital vitamins and minerals that make human life and survival possible in this part of the world.

### Mercury and selenium exposure

A strong positive relation between the concentrations of Hg and Selenium (Se) in tissues of many fish-eating wildlife species, especially predatory marine mammals, is well documented (Koeman et al. 1975). The Hg–Se relationship is a toxicant–nutrient interaction that has relevance for both basic biology and environmental risk assessment. High trophic level mammals and birds may be partially protected against MeHg toxicity due to chelation of inorganic Hg with Se in an approximate 1:1 molar ratio, respectively (Dietz et al. 2000a, 2013c). Hg and Se molar relationships in muscle, liver, and kidney tissue have been surveyed for a large number of Greenland marine animal species, showing that in a majority of the investigated individuals, Se was present in a molar surplus to Hg. A similar surplus of Se was found in recently collected narwhal tissues from the region in 2015, where Se:Hg molar ratios of 1.5, 2.3, and 17 were found for muscle, liver, and *mattak*, respectively. A 1:1 molar ratio was found in tissues of marine mammals with high Hg concentrations (above approx. 10 nmol g^−1^) in Greenland (AMAP 1998; Dietz et al. 1990, 1998, 2000a, b). These findings again support that MeHg is detoxified by a chemically inert Hg–Se (tiemanite) complex found in marine mammals (Koeman et al. 1975; Scheuhammer et al. 1998, 2008; Woshner et al. 2001a, b, 2008; O’Hara and Becker 2003; Ikemoto et al. 2005). The large surplus of Se in many wildlife tissues including the narwhal *mattak* is hence likely to protect the Inughuit against their high Hg exposure. A large proportion of the mattak is, however, being traded to Royal Greenland and Inughuit Fisheries and exported to the capital and larger Greenlandic towns, where *mattak* is not as readily available from the local hunt.

A substantial amount of information on the healthy nutrients, fatty acids, and vitamins is available, but a thorough description on these themes would remove the focus on the Hg issue of this article. The AMAP (2009) Human Health Assessment hence concluded and confirmed the ‘Arctic Dilemma’ described first in the second AMAP assessment of human health in the Arctic (AMAP 2003) that: “Traditional food is nutritionally, culturally, economically, and spiritually important but is also the major source of exposure to POPs and metals. Nevertheless, the results of this assessment support the importance of promoting the consumption of traditional food after providing information which allows informed choice.*”*

## Conclusion and perspectives for the future of the north water

The previous ways of evaluating temporal contaminant trends in Arctic wildlife and Inuit food items have focused on monitoring with regular time intervals a few “essential” species of ecological importance to the hunting communities. In addition, several studies on geographical and temporal contaminant trends have been conducted on the Inuit populations across the Arctic (AMAP 2003, 2009, 2015). However, the present study has, for the first time, estimated seasonal and long-term temporal change of Hg entering an Arctic community, i.e., Avanersuaq, based on a 20-year record of hunting trends as well as evaluation of the Hg content in a large number of important hunted foods. The results of this study detect the main sources of Hg from the hunt, of which the narwhal meat are the most important, and reveals a number of climate related as well as cultural and socio-economic changes affecting the invisible threat of Hg exposure in Inuit populations. This study add evidence to that long-range transport of Hg in even a remote hunting society like the Inughuit in Avanersuaq are heavily affected by anthropogenic processes from the industrialized part of the world. The food chain biomagnification of Hg renders marine top predators, being important food items for the NOW Inuit population, ultimate sinks of Hg amplify the effects of Hg from southern latitudes. However, the marine food has beneficial nutritional aspects as it is also rich in vitamins, micronutrients, fatty acids, and Se, which is particularly true for *mattak*. In fact, Se is likely an antagonistic mechanism reducing the toxic impact of Hg. Nonetheless, dietary advice in Greenland can reduce the exposure to Hg, and as such, the Greenland Board of Nutrition is tasked with providing balanced information to the public about contaminants in traditional marine food items and general information about a healthy and nutritious diet. Pregnant and nursing women, as well as children and young people are encouraged to continue to eat the traditional marine food but to avoid or reduce consumption of older seals, toothed whales, seabirds, and polar bear, due to high concentrations of contaminants. Supplementary hunt oriented towards more terrestrial animals and increased fishing on lower trophic species has the potential of reducing human exposure to Hg. Similar dietary advice is already given in other (sub)Arctic areas including Canada and the Faroe Islands, where consumption of marine mammals is also traditional. Finally, the Minamata Convention has recently entered into force, partly prompted by the documentation of high Hg concentrations within the Arctic marine ecosystem and hunting societies. It is the hope that it will facilitate a reduction of long-range transport of Hg into the Arctic similar to what has been achieved with the Stockholm Convention on POPs (Minamata Convention 2017; Stockholm Convention 2017).

### Recommendations

The calculated estimated Hg exposures should be verified by linking individual hunting success to the Hg exposure monitored in blood and hair in future programs. In addition, longitudinal health studies of Hg and human gene-environment studies in Greenland are still lacking. Finally, information on seasonal variation in human exposure should be taken into account when conducting geographical comparisons across the Arctic.

## Electronic supplementary material

Below is the link to the electronic supplementary material.

Supplementary material 1 (PDF 373 kb)
