# Supplementary material for: Interactions of climate, socio-economics, and global mercury pollution in the North Water
Source: Ambio. 2018 Mar 7;47(Suppl 2):281–95. doi: 10.1007/s13280-018-1033-z (PMC5963567; doi:10.1007/s13280-018-1033-z)
Supplement: Supplementary file 1 — Supplementary material 1 (PDF 373 kb) [file 13280_2018_1033_MOESM1_ESM.pdf]

Ambio

Electronic Supplementary Material

This supplementary material has not been peer reviewed.

Title: **Interactions of climate, socio-economics and global mercury pollution in the North Water region**

Authors: Rune Dietz, Anders Mosbech, Janne Flora and Igor Eulaers

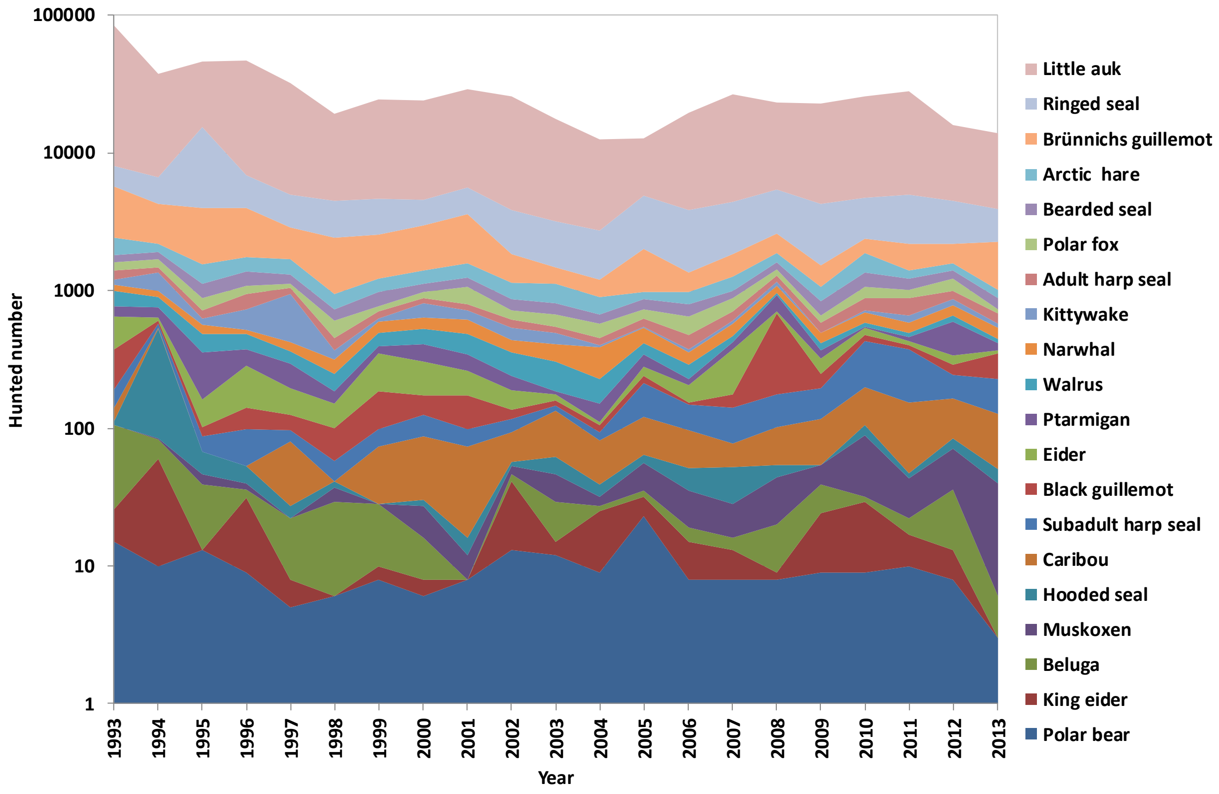

Fig. S1 *Yearly number of wildlife prey obtained from the aboriginal hunt in Avanersuaq between 1983 and 2013. Exact numbers are provided in SI Table 1. Source: Piniarneq, Greenland Home Government.*

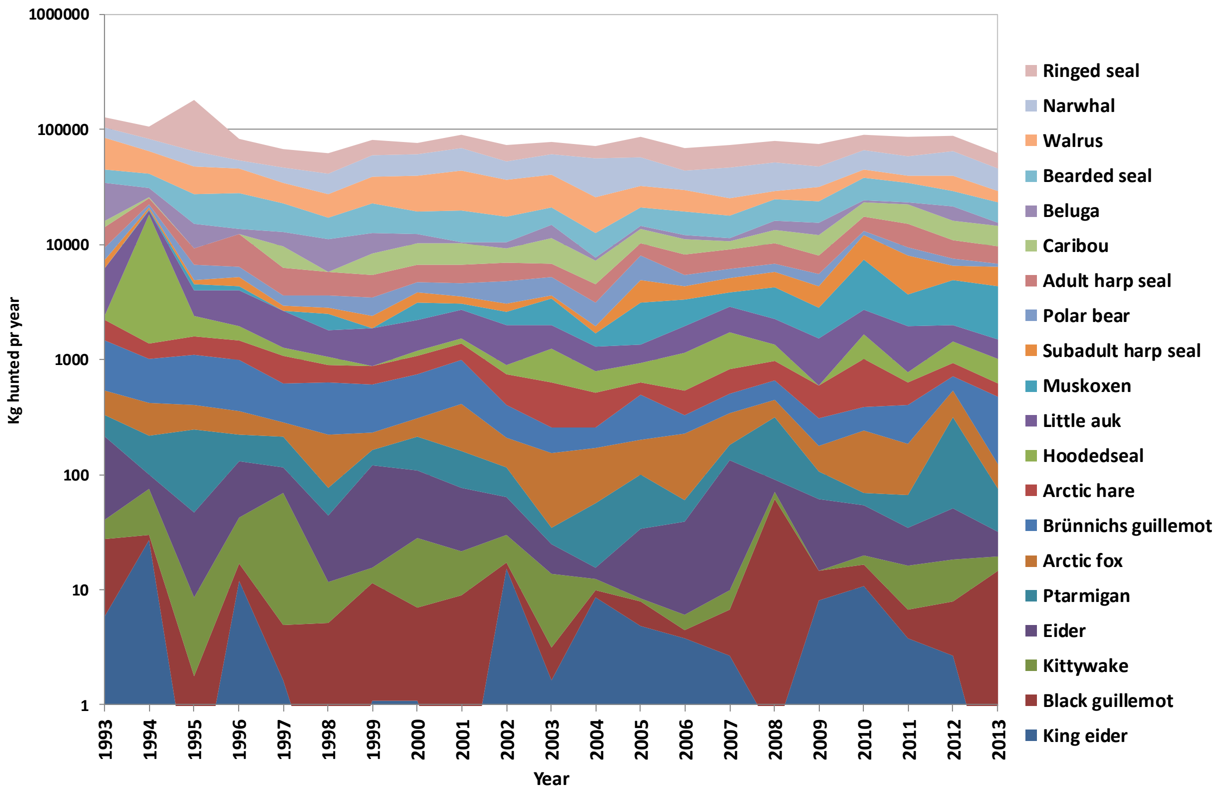

Fig. S2 *Estimated yearly amounts of meat (Kg) obtained from the aboriginal hunt in Avanersuaq between 1983 and 2013. Exact numbers are provided in SI Table 2. Figures calculated from the hunt information of Piniarneq, Greenland Home Government.*

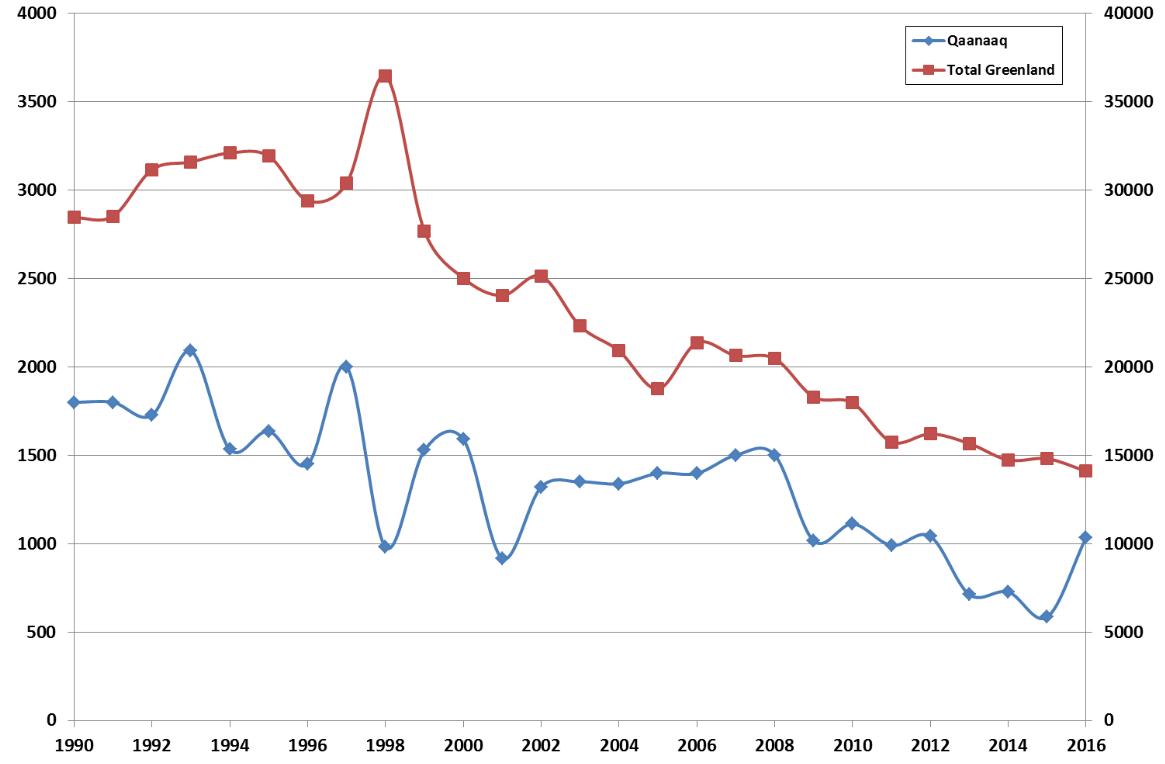

Fig S3 *Decline in* ***s****led dog numbers in the NOW region (left Y-axis) as well as in the entire Greenland (right Y-axis) based on information from Greenland Statistics.*

Table S1 *Temporal number of hunted game of Avanersuaq, NW Greenland based from 1993 to 2013 (Piniarneq 2016).*

Species\Year	1993	1994	1995	1996	1997	1998	1999	2000	2001	2002	2003	2004	2005	2006	2007	2008	2009	2010	2011	2012	2013	Average
Polar bear	15	10	13	9	5	6	8	6	8	13	12	9	23	8	8	8	9	9	10	8	3	10
King eider	11	50	0	22	3	0	2	2	0	28	3	16	9	7	5	1	15	20	7	5	0	10
Beluga	79	23	26	5	14	23	18	8	0	5	14	2	3	4	3	11	15	3	5	23	3	14
Mosk oxen	0	1	7	4	0	8	0	11	4	7	17	5	21	16	12	24	15	56	21	35	34	14
Hooded seal	7	444	21	13	5	4	0	3	4	4	16	7	8	16	24	10		17	4	13	10	32
Caribou	29	3	0	0	53	0	46	57	57	36	72	43	56	45	26	48	62	93	107	80	78	47
Black guillemot	182	24	14	43	28	42	87	49	74	19	13	11	26	6	34	503	54	49	25	44	120	69
Eider	278	39	61	143	72	52	166	129	87	54	18	5	40	53	198	30	75	55	29	52	20	79
Ptarmigan	109	117	196	89	96	32	44	103	81	50	9	40	66	20	48	222	43	15	32	257	43	82
Walrus	233	140	122	110	72	62	97	122	142	116	118	79	67	63	45	27	46	37	33	61	34	87
Narwhal	105	90	88	37	58	71	104	105	128	81	100	155	127	68	109	114	82	110	94	127	85	97
Kittywake	105	364	54	202	522	53	33	169	103	102	85	19	4	13	25	85	1	26	77	82	40	103
Harp seal	234	165	115	271	115	96	97	113	103	104	70	63	172	156	170	204	169	390	438	204	206	174
Polar fox	215	206	166	140	74	152	68	96	265	98	126	121	104	173	166	134	77	181	124	235	50	141
Bearded seal	213	209	238	287	195	122	210	147	192	143	129	97	132	151	127	173	167	283	215	159	159	178
Polar hare	612	307	420	385	383	219	234	278	316	279	308	216	119	176	265	267	237	525	186	183	121	287
Brünnichs guillem	3,262	2,088	2,448	2,236	1,163	1,461	1,313	1,550	2,016	690	365	303	1,027	367	571	746	445	500	766	628	1,246	1,200
Ringed seal	2,293	2,329	11,429	2,927	2,122	2,052	2,114	1,629	2,017	2,038	1,699	1,518	2,899	2,505	2,596	2,784	2,757	2,347	2,781	2,303	1,642	2,704
Little auk	75,712	30,616	30,671	39,830	27,104	14,684	19,762	19,577	23,454	21,861	14,517	9,782	7,772	15,687	21,943	17,777	18,677	20,886	23,042	11,335	9,895	22,599
Total game	83,694	37,225	46,089	46,753	32,084	19,139	24,403	24,154	29,051	25,728	17,691	12,491	12,675	19,534	26,375	23,168	22,946	25,602	27,996	15,834	13,789	27,925

Table S2 *Temporal estimated meet intake from hunted game of Avanersuaq, NW Greenland based from 1993 to 2013 (Piniarneq 2016).*

Species\Year	1993	1994	1995	1996	1997	1998	1999	2000	2001	2002	2003	2004	2005	2006	2007	2008	2009	2010	2011	2012	2013	Mean
King eider	5.9	27.0	0.1	11.9	1.6	0.1	1.1	1.1	0.1	15.1	1.6	8.6	4.9	3.8	2.7	0.5	8.1	10.8	3.8	2.7	0.1	5.3
Black guillemot	21.8	2.9	1.7	5.2	3.4	5.0	10.4	5.9	8.9	2.3	1.6	1.3	3.1	0.7	4.1	60.4	6.5	5.9	3.0	5.3	14.4	8.3
Kittywake	13.1	45.3	6.7	25.1	65.0	6.6	4.1	21.0	12.8	12.7	10.6	2.4	0.5	1.6	3.1	10.6	0.1	3.2	9.6	10.2	5.0	12.8
Eider	175.1	24.6	38.4	90.1	45.4	32.8	104.6	81.3	54.8	34.0	11.3	3.2	25.2	33.4	124.7	18.9	47.3	34.7	18.3	32.8	12.6	49.7
Ptarmigan	111.2	119.3	199.9	90.8	97.9	32.6	44.9	105.1	82.6	51.0	9.2	40.8	67.3	20.4	49.0	226.4	43.9	15.3	32.6	262.1	43.9	83.2
Polar fox	206.4	197.8	159.4	134.4	71.0	145.9	65.3	92.2	254.4	94.1	121.0	116.2	99.8	166.1	159.4	128.6	73.9	173.8	119.0	225.6	48.0	135.8
Brünnichs guillem	929.7	595.1	697.7	637.3	331.5	416.4	374.2	441.8	574.6	196.7	104.0	86.4	292.7	104.6	162.7	212.6	126.8	142.5	218.3	179.0	355.1	341.9
Polar hare	734.4	368.4	504.0	462.0	459.6	262.8	280.8	333.6	379.2	334.8	369.6	259.2	142.8	211.2	318.0	320.4	284.4	630.0	223.2	219.6	145.2	344.9
Hooded seal	268.3	17,020.0	805.0	498.3	191.7	153.3	0.1	115.0	153.3	153.3	613.3	268.3	306.7	613.3	920.0	383.3	0.1	651.7	153.3	498.3	383.3	1,150.0
Little auk	3,861.3	1,561.4	1,564.2	2,031.3	1,382.3	748.9	1,007.9	998.4	1,196.2	1,114.9	740.4	498.9	396.4	800.0	1,119.1	906.6	952.5	1,065.2	1,175.1	578.1	504.6	1,152.6
Musk oxen	0.1	84.0	588.0	336.0	0.1	672.0	0.1	924.0	336.0	588.0	1,428.0	420.0	1,764.0	1,344.0	1,008.0	2,016.0	1,260.0	4,704.0	1,764.0	2,940.0	2,856.0	1,192.0
Polar bear	2,025.0	1,350.0	1,755.0	1,215.0	675.0	810.0	1,080.0	810.0	1,080.0	1,755.0	1,620.0	1,215.0	3,105.0	1,080.0	1,080.0	1,080.0	1,215.0	1,215.0	1,350.0	1,080.0	405.0	1,285.7
Harp seal	5,900.0	4,106.7	2,933.3	6,920.0	2,960.0	2,446.7	2,420.0	2,760.0	2,573.3	2,613.3	1,786.7	1,600.0	3,973.3	3,813.3	4,113.3	4,940.0	3,986.7	8,866.7	10,226.7	4,906.7	4,820.0	4,222.2
Rensdyr	1,870.5	193.5	0.1	0.1	3,418.5	0.1	2,967.0	3,676.5	3,676.5	2,322.0	4,644.0	2,773.5	3,612.0	2,902.5	1,677.0	3,096.0	3,999.0	5,998.5	6,901.5	5,160.0	5,031.0	3,043.8
Beluga	18,433.3	5,366.7	6,066.7	1,166.7	3,266.7	5,366.7	4,200.0	1,866.7	0.1	1,166.7	3,266.7	466.7	700.0	933.3	700.0	2,566.7	3,500.0	700.0	1,166.7	5,366.7	700.0	3,188.9
Bearded seal	10,650.0	10,450.0	11,900.0	14,350.0	9,750.0	6,100.0	10,500.0	7,350.0	9,600.0	7,150.0	6,450.0	4,850.0	6,600.0	7,550.0	6,350.0	8,650.0	8,350.0	14,150.0	10,750.0	7,950.0	7,950.0	8,923.8
Walrus	38,833.3	23,333.3	20,333.3	18,333.3	12,000.0	10,333.3	16,166.7	20,333.3	23,666.7	19,333.3	19,666.7	13,166.7	11,166.7	10,500.0	7,500.0	4,500.0	7,666.7	6,166.7	5,500.0	10,166.7	5,666.7	14,492.1
Narwhal	21,000.0	18,000.0	17,600.0	7,400.0	11,600.0	14,200.0	20,800.0	21,000.0	25,600.0	16,200.0	20,000.0	31,000.0	25,400.0	13,600.0	21,800.0	22,800.0	16,400.0	22,000.0	18,800.0	25,400.0	17,000.0	19,409.5
Ringed seal	22,930.0	23,290.0	114,290.0	29,270.0	21,220.0	20,520.0	21,140.0	16,290.0	20,170.0	20,380.0	16,990.0	15,180.0	28,990.0	25,050.0	25,960.0	27,840.0	27,570.0	23,470.0	27,810.0	23,030.0	16,420.0	27,038.6
Total	127,969.6	106,135.9	179,443.5	82,977.5	67,539.6	62,253.2	81,167.1	77,205.8	89,419.5	73,517.2	77,834.6	71,957.0	86,650.4	68,728.3	73,051.1	79,757.1	75,490.9	90,003.8	86,225.1	88,013.7	62,360.9	86,081.0

***Table S3** Mercury concentration in game of Northwest Greenland based on published literature and the present study.*

Species	Hg Conc (ppm)	Region	Year	n	Reference
Marine birds					
Black guillemot	0.370	Avanersuaq	1984	26	Overgaard & Dietz 1989
	0.270	Avanersuaq	2015	10	This study
	0.320	Avanersuaq	1984-2015	36	Value used in calculations
Brünnichs guillemot	0.300	Avanersuaq	1984	15	Overgaard & Dietz 1989
	0.296	Avanersuaq	2015	11	This study
	0.298	Avanersuaq	1984-2015	26	Value used in calculations
Common eider	0.150	West Greenland	1984	20	Overgaard & Dietz 1989
	0.098	Avanersuaq	2015	3	This study
	0.124	Avanersuaq	1984-2015	3	Value used in calculations
Fulmar	0.470	Avanersuaq	1984	14	Overgaard & Dietz 1989
	0.345	Avanersuaq	2015	11	This study
	0.407	Avanersuaq	1984-2015	25	Value used in calculations
Glaucous gull	0.940	Avanersuaq	1984	11	Overgaard & Dietz 1989
	0.588	Avanersuaq	2015	11	This study
	0.764	Avanersuaq	1984-2015	22	Value used in calculations
King eider	0.130	West Greenland	1982	21	Overgaard & Dietz 1989
		Avanersuaq	to be sampled 2017	20	This study
Kittywake	0.390	Avanersuaq	1984	14	Overgaard & Dietz 1989
	0.148	Avanersuaq	2015	11	This study
	0.269	Avanersuaq	1984-2015	25	Value used in calculations
Little auk	0.110	Avanersuaq	1984	25	Overgaard & Dietz 1989
	0.075	Avanersuaq	2015	10	This study
	0.092	Avanersuaq	1984-2015	35	Value used in calculations
Terrestrial birds					
Ptarmigan	0.005	Avanersuaq	1999	10	Johansen et al 2004
Terrestrial mammals					
Caribou	0.013	Avanersuaq	1995-1997	127	Astrup et al. 2000
Muskoxen	0.005	West Greenland	1999	29	Johansen 2004
Polar fox	0.051	Victoria Island	1973	7	Smith & Armstrong 1975
Arctic hare	0.002	West Greenland	1999	5	Johansen 2004
Marine mammals					
Bearded seal	0.530	Amundsen Gulf	1973	3	Smith & Armstrong 1975
	0.090	Belcher Islands	1974	55	Smith & Armstrong 1975
	0.310	Canada	1973-74	58	Value used in calculations
Harp seal all age groups	0.228	Nuuk	2001	28	Johansen 2005
Harp seal juvenile	0.290	Davis Strait	1998-2009	21	Dietz et al. in press
Harp seal adult	0.450	Davis Strait	1998-2009	21	Dietz et al. in press
Harp seal all age groups	0.323	West Greenland	1973-2009	70	Value used in calculations
Hooded seals 2-4y	0.485	Upernavik	1985	1	Dietz et al. 1998
Hooded seals >15	0.812	Upernavik	1985	2	Dietz et al. 1998
Hooded seal juvenile	0.210	Davis Strait	2002-2008	21	Dietz et al. in press
Hooded seal adult	0.610	Davis Strait	2002-2008	21	Dietz et al. in press
Hooded seal all age groups	0.548	West Greenland		45	Value used in calculations
Walrus	0.057	Avanersuaq	1975-77	9	Born et al. 1981
Beluga	0.281	Avanersuaq	1984-1985	41	Hansen et al 1990
Narwhal males	1.820	Avanersuaq	1984	11	Dietz et al. 2004
	0.707	Avanersuaq	1985	12	Dietz et al. 2004
Narwhal females	1.070	Avanersuaq	1984	25	Dietz et al. 2004
	0.662	Avanersuaq	1985	5	Dietz et al. 2004
	1.190	Avanersuaq	1993	3	Dietz et al. 2004
	1.340	Avanersuaq	2015	10	This study
Narwhal all	1.132	Avanersuaq	1984-2015	66	Value used in calculations
Ringed seal	0.241	Avanersuaq		14	Dietz et al. 1998
Polar bear	0.057	Avanersuaq		36	Dietz et al. 2000

***Table S4** Temporal yearly influx of Hg from the hunted game of Avanersuaq based on average hunt from 1993 to 2013 (Piniarneq 2016).*

Species\Year	1993	1994	1995	1996	1997	1998	1999	2000	2001	2002	2003	2004	2005	2006	2007	2008	2009	2010	2011	2012	2013	Average
Polar hare	0.0015	0.0007	0.0010	0.0009	0.0009	0.0005	0.0006	0.0007	0.0008	0.0007	0.0007	0.0005	0.0003	0.0004	0.0006	0.0006	0.0006	0.0013	0.0004	0.0004	0.0003	0.0007
Ptarmigan	0.0022	0.0024	0.0040	0.0018	0.0020	0.0007	0.0009	0.0021	0.0017	0.0010	0.0002	0.0008	0.0013	0.0004	0.0010	0.0045	0.0009	0.0003	0.0007	0.0052	0.0009	0.0017
Musk ox		0.0004	0.0029	0.0017	0.0025	0.0034	0.0040	0.0046	0.0017	0.0029	0.0071	0.0021	0.0088	0.0067	0.0050	0.0101	0.0063	0.0235	0.0088	0.0147	0.0143	0.0066
Arctic fox	0.0105	0.0101	0.0081	0.0069	0.0036	0.0074	0.0033	0.0047	0.0130	0.0048	0.0062	0.0059	0.0051	0.0085	0.0081	0.0066	0.0038	0.0089	0.0061	0.0115	0.0024	0.0069
Caribou	0.0187	0.0019	0.0140	0.0241	0.0342	0.0319	0.0297	0.0368	0.0368	0.0232	0.0464	0.0277	0.0361	0.0290	0.0168	0.0310	0.0400	0.0600	0.0690	0.0516	0.0503	0.0338
Polar bear	0.1154	0.0770	0.1000	0.0693	0.0385	0.0462	0.0616	0.0462	0.0616	0.1000	0.0923	0.0693	0.1770	0.0616	0.0616	0.0616	0.0693	0.0693	0.0770	0.0616	0.0231	0.0733
Little auk	0.2885	0.1167	0.1169	0.1518	0.1033	0.0560	0.0753	0.0746	0.0894	0.0833	0.0553	0.0373	0.0296	0.0598	0.0836	0.0677	0.0712	0.0796	0.0878	0.0432	0.0377	0.0861
Marine birds - LA	0.3011	0.1907	0.2117	0.2046	0.1134	0.1288	0.1246	0.1436	0.1797	0.0667	0.0357	0.0296	0.1330	0.0478	0.0904	0.0864	0.0464	0.0641	0.0693	0.0725	0.1118	0.1168
Hooded seal	0.2200	13.9564	0.6601	0.4086	0.1572	0.1257	0.1100	0.0943	0.1257	0.1257	0.5029	0.2200	0.2515	0.5029	0.7544	0.3143	0.4244	0.5344	0.1257	0.4086	0.3143	0.9684
Harp seal	1.3670	0.9639	0.6718	1.5831	0.6718	0.5608	0.5667	0.6601	0.6017	0.6075	0.4089	0.3680	1.0048	0.9113	0.9931	1.1917	0.9873	2.2783	2.5587	1.1917	1.2034	1.0167
Beluga	6.6597	1.9389	2.1918	0.4215	1.1802	1.9389	1.5174	0.6744	0.5480	0.4215	1.1802	0.1686	0.2529	0.3372	0.2529	0.9273	1.2645	0.2529	0.4215	1.9389	0.2529	1.1782
Walrus	6.5240	3.9200	3.4160	3.0800	2.0160	1.7360	2.7160	3.4160	3.9760	3.2480	3.3040	2.2120	1.8760	1.7640	1.2600	0.7560	1.2880	1.0360	0.9240	1.7080	0.9520	2.4347
Bearded seal	3.8068	3.7353	4.2536	5.1294	3.4851	2.1804	3.7532	2.6272	3.4315	2.5558	2.3055	1.7336	2.3592	2.6987	2.2698	3.0919	2.9847	5.0579	3.8426	2.8417	2.8417	3.1898
Ringed seal	5.5261	5.6129	6.3335	7.0541	5.1140	4.9453	5.0947	3.9259	4.8610	4.9116	4.0946	3.6584	6.9866	6.0371	6.2564	6.7094	6.6444	5.6563	6.7022	5.5502	3.9572	5.5063
Narhval	36.3825	31.1850	30.4920	12.8205	20.0970	24.6015	36.0360	36.3825	44.3520	28.0665	34.6500	53.7075	44.0055	23.5620	37.7685	39.5010	28.4130	38.1150	32.5710	44.0055	29.4525	33.6270
Sum	61.5253	61.9031	48.6752	31.1387	33.1305	36.4604	50.1045	48.2372	57.9121	40.2859	46.7258	62.2710	57.2606	36.0753	49.9126	52.8467	41.8665	53.3017	47.5341	57.9780	39.3267	48.3082
Hg PTWI (60 Kg) (mg)	0.1	0.1	0.1	0.1	0.1	0.1	0.1	0.1	0.1	0.1	0.1	0.1	0.1	0.1	0.1	0.1	0.1	0.1	0.1	0.1	0.1	0.1
Hg PTYI (60 Kg) (mg)	5.0	5.0	5.0	5.0	5.0	5.0	5.0	5.0	5.0	5.0	5.0	5.0	5.0	5.0	5.0	5.0	5.0	5.0	5.0	5.0	5.0	5.0
Population number	868	876	881	879	870	875	857	864	875	854	859	859	850	865	846	831	802	795	821	796	801	849
NOW Hg PTYI (g)	4.3	4.4	4.4	4.4	4.3	4.4	4.3	4.3	4.4	4.3	4.3	4.3	4.2	4.3	4.2	4.1	4.0	4.0	4.1	4.0	4.0	4.2
Sum Hg Influx/PTYI	14.2	14.2	11.1	7.1	7.6	8.3	11.7	11.2	13.3	9.4	10.9	14.5	13.5	8.4	11.8	12.7	10.5	13.4	11.6	14.6	9.8	11.4

Table S5 *Seasonal Hg load in the hunted game of Avanersuaq based on average hunt from 1994 to 2014 (Piniarneq 2016) and average Hg loads in muscle tissue from published and unpublished contaminant studies in Greenland.*

Species\Month	1	2	3	4	5	6	7	8	9	10	11	12
**Polar hare**	0.00002	0.00009	0.00007	0.00013	0.00001	0.00000	0.00000	0.00001	0.00009	0.00006	0.00003	0.00004
**Hooded seal**	0.00000	0.00629	0.00000	0.01572	0.00000	0.04401	0.04401	0.03143	0.10687	0.09116	0.00314	0.00000
**Other birds**	0.00013	0.00020	0.00024	0.00037	0.00789	0.02190	0.01071	0.00151	0.00258	0.00180	0.00002	0.00001
**Little auk**	0.00000	0.00000	0.00021	0.00000	0.02912	0.05339	0.02602	0.00211	0.00003	0.00107	0.00000	0.00000
**Polar bear**	0.00385	0.01154	0.01385	0.02309	0.00693	0.00770	0.00077	0.00000	0.00462	0.00077	0.00000	0.00000
**Bedlamer**	0.00680	0.00185	0.00124	0.00062	0.00124	0.00803	0.01421	0.05068	0.27686	0.23175	0.01792	0.00742
**Musk oxen**	0.00000	0.00000	0.00071	0.00109	0.00000	0.00004	0.00004	0.00319	0.00344	0.00147	0.00004	0.00000
**Saddleback**	0.01392	0.00619	0.00077	0.00851	0.00619	0.01083	0.02475	0.12373	0.40445	0.26680	0.02861	0.01083
**Harp seal**	0.02072	0.00804	0.00201	0.00912	0.00742	0.01886	0.03896	0.17441	0.68132	0.49855	0.04654	0.01824
**Caribou**	0.00000	0.00013	0.00555	0.00277	0.00052	0.00006	0.00006	0.01387	0.01290	0.00529	0.00000	0.00000
**Walrus**	0.00667	0.01867	0.05067	0.05333	0.11200	0.05867	0.01733	0.00533	0.01600	0.28000	0.03600	0.00133
**Bearded seal**	0.08215	0.13250	0.11395	0.12985	0.39485	0.84535	0.59625	0.59890	0.78705	0.62540	0.06625	0.03445
**Narwhal**	0.01126	0.06756	0.11260	0.05630	0.34906	2.71366	3.47934	3.96352	0.75442	0.47292	0.05630	0.02252
**Ringed seals**	0.47236	0.36222	0.23401	0.26558	0.48730	0.43211	0.26414	0.44947	1.04136	0.69432	0.52996	0.58298
**Total**	0.61854	0.61601	0.53672	0.56696	1.41041	4.23653	4.52731	5.41988	4.09562	3.17508	0.78601	0.67837
**NOW Hg PTMI (g)**	0.32592	0.32592	0.32592	0.32592	0.32592	0.32592	0.32592	0.32592	0.32592	0.32592	0.32592	0.32592
**Sum Hg Influx/PTMI**	1.89780	1.89003	1.64676	1.73954	4.32742	12.99850	13.89066	16.62924	12.56616	9.74177	2.41162	2.08138
